# Determinants of early initiation of breastfeeding in Nigeria: a population-based study using the 2013 demograhic and health survey data

**DOI:** 10.1186/s12884-016-0818-y

**Published:** 2016-02-06

**Authors:** Anselm S. Berde, Siddika Songül Yalcin

**Affiliations:** Institute of Public Health, Hacettepe University, Ankara, Turkey; Department of Social Peadiatrics, Hacettepe University, Ankara, Turkey

**Keywords:** Initiation of breastfeeding, Nigeria, Mothers, Infants

## Abstract

**Background:**

Provision of mother’s breast milk to infants within one hour of birth is referred to as Early Initiation of Breast Feeding (EIBF) which is an important strategy to reduce perinatal and infant morbidities and mortality. This study aimed to use recent nationally representative survey data to identify individual, household and community level factors associated with EIBF and to update on previous knowlegde with regards to EIBF in Nigeria.

**Methods:**

We used cross-sectional data from the 2013 Nigerian Demographic and Health Survey (NDHS). Chi-square tests and binary logistic regression were used to test for association between EIBF and individual, household and community level factors.

**Result:**

The proportion of infants who initiated breastfeeding within 1 h of birth was 34.7 % (95 % Confidence Interval (CI): 33.9–35.6). In the multivariate analysis, mothers who delivered in a health facility were more likely to initiate breastfeeding early as compared to mothers who delivered at home (Adjusted Odds Ratio (AOR) =1.40, 95 % CI = 1.22–1.60). The odds of EIBF was three times higher for mothers who had vaginal delivery as compared to mothers who had caesarean section (AOR = 3.08, 95 % CI = 2.14–4.46). Other factors that were significantly associated with increased likelihood of EIBF were; multiparity, large sized infant at birth, not working mothers as compared to mothers working in sales and other sectors, wealthier household index and urban residence. Mothers in the South West were less likely to inititiate breastfeeding within 1 h of birth as compared to the North West, however, the following geopolitical zones; North East, North Central, and South South had higher likelihood of EIBF when compared to the North West geopolitical zone.

**Conclusion:**

EIBF in Nigeria is not optimal with just about 34.7 % of children initiating breastfeeding within one hour of birth, the results suggest that breastfeeding programmes and policies should give special attention to “rural mothers, working mothers, primiparous mothers, mothers with ceasarean deliveries, home deliveries and poor mothers” and this intervention should cut across geopolitical zones with more emphasis to zones with lower rates of EIBF.

## Background

Breastfeeding is a foundation practice for appropriate care and feeding of newborn infants [1] and has nutritional, immunological, developmental, psychological, social, economic and environmental benefits for infants, mothers, families and society [2]. Provision of mother’s breast milk to infants within one hour of birth is referred to as “Early Initiation of Breast Feeding (EIBF)” [3]. This practice ensures that the infant receives colostrum which is rich in immunoglobulin (Ig) and other bioactive molecule, including growth factors that are important for nutrition, growth and development of newborn infants and also for passive immunity [4]. Clemens et al. [5] in a cohort study done in rural Egypt found that EIBF was associated with marked reduction in the rate of diarrhea throughout the first 6 months of life possibly because of the salutary effects of human colostrum. Furthermore, EIBF enhances “Maternal-infant Bonding” defined as the development of the core relationship between mother and child which begins in early infancy and continues over the next few years with tremendous implications for the child’s future development [6]. In addition, infants breastfed within 30 min of birth are likely to remain breast fed for a longer period of time [7]. Research findings in Ghana and Nepal, showed that approximately one-fifth of all neonatal deaths could be avoided if breastfeeding were initiated within the first hour of life for all newborns [8, 9]. Furthermore, EIBF is also beneficial to mothers as it decreases postpartum bleeding and is associated with rapid uterine involution due to increase concentrations of oxytocin [10].

Over the years, the United Nations Children’s Fund (UNICEF) and World Health Organisation (WHO) have promoted EIBF as an important strategy to reduce perinatal and infant morbidities and mortality through programmes such as the Baby Friendly Hospital Initiative (BFHI), Community Integrated Management of Childhood Illness (C-IMCI) and Infant and Young Child Feeding (IYCF) [3, 10, 11]. In Nigeria, the Federal Ministry of Health in conjunction with UNICEF and WHO launched the BFHI in 1992 to protect, promote and support breastfeeding in Nigeria [12]. The initiative presumes that most mothers would come in contact with these specially designated hospitals and be exposed to better education on breastfeeding [13]. However, the reported pattern of maternal health service utilization in Nigeria is low [14] and this may serve as a major hindrance to the initiative. In addition to the BFHI, there are other programmes promoting EIBF in Nigeria such as the national policy on IYCF and the C-IMCI [15, 16]. UNICEF has rated the overall national IYCF policy, strategy and plan of action as fair [15].

Though, breastfeeding is almost universal in Nigeria, with 97.9 % of all children breastfed for a period of time [17], prevalence from national studies done in Nigeria report low figures for EIBF with just about 31.9 and 38.4 % of mothers initiating breastfeeding within 1 h of birth in 2003 and 2008 respectively [18, 19].

Previous studies have shown that EIBF is multi-factorial in nature and involves factors such as mother’s age, education, place of residence and health service utilization such as antenatal care (ANC) visits and place of delivery [3, 12, 13, 20, 21]. In Nigeria, there is a need for a more detailed understanding of the factors associated with EIBF at the national level, so as to help design programs aimed at increasing breastfeeding initiation within 24 h of birth. This study aimed to use recent nationally representative survey data to identify individual, household and community level factors associated with EIBF and to update on previous knowledge with regards to EIBF in Nigeria.

## Methods

In this study, we used data from the 2013 Nigerian Demographic and Health Survey (NDHS). It is the fifth and most recent in the series of Demographic and Health Surveys conducted so far in Nigeria. The 2013 NDHS sample was selected using a stratified three-stage cluster design consisting of 904 clusters, 372 in urban areas and 532 in rural areas. Further details of the sampling techniques and data collection method can be found in the DHS manual [17]. Analysis for this study was restricted to mothers with last-born children born in the past two years preceeding the survey and the total sample size was 11851. After accounting for sample weights, this corresponded to a sample size of 11910.

### Definition of variables

In the NDHS woman’s questionnaire, mothers were asked “How long after birth did you first put (NAME) to the breast?” Responses were recorded in number of hours or days [17]. Our outcome variable “EIBF” was defined as initiation of breastfeeding within 1 h of birth and was expressed as a dichotomous variable with category 1 for initiation of breastfeeding within 1 h (early) and category 0 for initiation of breastfeeding after 1 h (late). The explanatory factors were choosen based on previous studies [12, 13, 20, 21] and grouped into individual, household and community level characteristics. Some of the variables were recoded while others were adopted as reported in the 2013 NDHS. Explanatory variables included ungrouped mothers age at birth recoded into “<=19”, “20–24”, “25–29”, “30–34” and “> = 35” years. Mothers education was categorized as “no education”, “primary”, “secondary and above”. Birth order was recoded into “1st”, “2nd–3rd” and “4th and above birth order”. Number of ANC visit was recoded into “0”, “1–3”, “4 and above visit”. Place of delivery was categorized as “home” and “health facility”. Also considered was mode of delivery (“normal” or “caesarean”). Sex of child was as reported in the 2013 NDHS (“male”-“female”). Size of child at birth based on mothers perception was categorized into three groups namely; “large”, “average” and “small”. Birth type was recoded into “singleton” or “twin/multiple” while mothers education was recoded into three groups; “no education”, “primary”, “secondary and above”. Mothers occupation was categorized into “not working”, “agricultural”, “sales and others”. DHS wealth index was as reported in 2013 NDHS (“poorest”, “poorer”, “middle”, “richer” and “richest”), the index was constructed using household asset data via a principal components analysis. Place of residence was as reported in the 2013 NDHS (“urban”-“rural”). In terms of zones, all the six geopolitical zones in the country were considered.

### Statistical analysis

Chi square tests were performed to evaluate the association of the independent variables with EIBF. Rate of EIBF and distribution by different independent variables were reported as weighted percentages and 95 % CI using Stata version 13 and then further assessed by binary logistic regression to examine the likely predictors of EIBF in Nigeria. Unadjusted and adjusted odds ratios (OR) with their 95 % confidence interval (CI) were reported. The multivariate analysis accounted for the sample design and sample weight using Statistical Package for Social Sciences (SPSS) complex sample analysis method [22] (SPSS version 21).

### Ethics

The study was a secondary analysis of freely available data, as such, no formal ethical clearance was required. Permission to use and analyse the dataset was obtained by registering the study (Project number 64273) on the Demographic and Health Survey (DHS) website.

## Results

### Socio-demographic characteristics of the mothers

A total of 11851 mothers with last born children within the past two years preceeding the survey were considered for the analysis. As summarized in Table 1, most mothers where within the age group 20–24 and 25–29 years at the time of birth (24.9 % and 27.3 % repectively). The highest proportion of mothers had no education (45.4 %) and the highest percentage of mothers (48.2 %) had 4th or higher order births. Over half (53.2 %) of mothers had at least 4 ANC visit. Of the total births, 38 % took place in a health facility. The highest proportion of deliveries (97.8 %) were vaginal and male and female children were more or less equal in the sample. 44.1 % of mothers percieved the size of their child at birth as large and the highest proportion of birth were singleton (98.3 %). Approximately 57.2 % of mothers were employed in sales and other sectors and about 23.2 % of mothers belonged to the poorer wealth quintile. The distribution also showed that a larger proportion of mothers (67 %) lived in rural area. Furthermore, the highest proportion of mothers were from the North West (31.2 %) and North East (20.4 %) geopolitical zones respectively.

**Table 1:** Individual, household, community level characteristics and rates (%) of initiation of breastfeeding within one hour of child birth, Nigeria 2013 (*N* = 11851)

Characteristics	Total		Initiation of breastfeeding within 1 h of birth			
	N^a^	%^b^	n ^c^	%^d^	95 % CI	*p* value^e^
Individual level factors						
Mother’s age at birth						
<=19	1661	14.0	526	30.3	(28.2–32.5)	<0.001
20–24	2953	24.9	1002	34.0	(32.3–35.7)	
25–29	3237	27.3	1190	36.3	(34.6–36.9)	
30–34	2119	17.9	772	36.8	(34.7–38.8)	
> = 35	1881	15.9	649	35.1	(32.9–37.2)	
Mothers education						
No education	5379	45.4	1702	30.0	(28.8–31.2)	<0.001
Primary	2290	19.3	789	36.8	(34.8–38.9)	
Secondary and above	4182	35.3	1647	40.2	(38.7–41.7)	
Birth order						
1st	2331	19.7	774	32.5	(30.6–34.4)	0.013
2nd–3rd	3807	32.1	1392	36.2	(34.7–37.7)	
4th and above	5713	48.2	1972	34.7	(33.5–35.9)	
ANC visit (*n* = 11553)						
0	3837	33.2	1083	27.6	(26.1–28.9)	<0.001
1–3	1573	13.6	580	36.8	(34.4–39.1)	
4+	6143	53.2	2343	38.3	(37.1–39.6)	
Place of delivery (*n* = 11846)						
Home	7339	62.0	2266	30.4	(29.4–31.5)	<0.001
Health facility	4507	38.0	1872	42.0	(40.5–43.4)	
Mode of delivery (n = 11771)						
Vaginal delivery	11508	97.8	4057	35.1	(34.2–35.9)	<0.001
Caesarean section	263	2.2	56	22.4	(17.1–27.4)	
Sex of child						
Male	6021	50.8	2076	34.7	(33.5–36.0)	0.985
Female	5830	49.2	2062	34.8	(33.5–36.0)	
Size of child at birth (*n* = 11791)						
Small	1811	15.4	536	29.5	(27.4–31.6)	<0.001
Average	4776	40.5	1608	33.3	(32.0–34.7)	
Large	5204	44.1	1977	37.9	(27.4–31.6)	
Birth type						
Singleton	11654	98.3	4081	34.9	(34.0–35.7)	0.046
Twin/mutiple	197	1.7	57	27.9	(21.8–34.1)	
Mothers occupation (*n* = 11789)						
Not working	3704	31.4	1368	36.6	(35.0–38.1)	<0.001
Agricultural	1341	11.4	461	37.6	(34.9–40.3)	
Sales and others	6744	57.2	2295	33.3	(32.2–34.4)	
Household level factors						
Wealth Index						
Poorest	2618	22.1	646	23.4	(21.8–25.0)	<0.001
Poorer	2754	23.2	849	31.3	(29.5–33.0)	
Middle	2368	20.0	854	37.9	(35.9–39.9)	
Richer	2207	18.6	932	43.5	(41.4–45.6)	
Richest	1904	16.1	858	41.9	(39.8–44.1)	
Community level factors						
Type of place of residence						
Urban	3908	33.0	1766	41.9	(40.4–43.4)	<0.001
Rural	7943	67.0	2372	30.8	(29.8–31.8)	
Zone						
North Central	1739	14.7	790	48.3	(45.8–50.7)	<0.001
North East	2420	20.4	816	39.9	(37.8–42.1)	
North West	3699	31.2	1171	26.9	(25.6–28.2)	
South East	1076	9.1	374	34.5	(31.7–37.4)	
South South	1431	12.1	506	45.1	(42.2–48.0)	
South West	1486	12.5	480	28.7	(26.5–30.8)	
Overall	11851	100.0	4138	34.7	(33.9–35.6)	

^a^Unweighted case numbers (the numbers and percentages reported are unweighted to facilitate reading as weighted count (frequency) will be in decimal points generated by the software). ^b^Unweighted column %, ^c^Weighted case numbers, ^d^ Weighted row %. ^e^Chi square test was applied to test statistical significance

### Results of bivariate analysis

A total of 4138 mothers, initiated breastfeeding within one hour of birth (weighted proportion 34.7 %; 95 % CI: 33.9–35.6). The bivariate analysis revealed that EIBF was significantly higher among mothers who delivered in a health facility (42 %) as compared to 30.4 % of mothers who delivered at home (*p* < 0.001). Also, mothers with 2nd–3rd and 4th and above birth order had significantly (*p* = 0.013) higher figures with regards to EIBF (36.2 and 34.7 % respectively) as compared to mothers with 1st birth (32.5 %). Increasing mothers age (*p* < 0.001), higher educational status (*p* < 0.001), increasing ANC visit (*p* < 0.001), vaginal delivery (*p* < 0.001), increasing size of child at birth (*p* < 0.001), singleton birth (*p* = 0.046), not working (*p* < 0.001), wealthier household wealth index (*p* < 0.001), urban residence (*p* < 0.001) and all zones as compared to the North Western zone (*p* < 0.001) were all significantly associated with higher EIBF rate (Table 1).

### Multivariate analysis

Results from the multivariate analysis (Table 2) indicated that place of delivery, mode of delivery, birth order, size of child at birth, mothers occupation, household wealth index, type of place of residence and zones were the determinants of EIBF. Mothers who delivered in a health facility were more likely to initiate breastfeeding within 1 h of birth as compared to mothers who delivered at home (Adjusted Odds Ratio (AOR) = 1.40, 95 % Confidence Interval (CI) =1.22–1.60). The odds of EIBF was 3 times higher for mothers who had vaginal delivery as compared to mothers who had caesarean section (AOR = 3.08, 95 % CI = 2.14–4.46). Likewise, the odds of EIBF was 1.19 times higher for mothers who perceived their infants to be large sized at birth as compared to mothers who perceived their infants to be small sized at birth (AOR = 1.19, 95 % CI = 1.03–1.39). Urban mothers were more likely to commence breastfeeding early as compared to their rural counterpart (AOR 1.46, 95 % CI = 1.23–1.75). Compared to the North Western zone, mothers who lived in the following geopolitical zones of Nigeria were significantly more likely to initiate breastfeeding within 1 h of birth: North East (AOR = 1.69, 95 % CI = 1.38–2.07); North Central (AOR = 1.93, 95 % CI = 1.56–2.39); and South South (AOR = 1.42, 95 % CI = 1.12–1.81); with the exception of the South West (AOR = 0.56, 95 % CI = 0.43–0.73). Furthermore, mothers with 1st birth had lower odds for EIBF as compared to mothers who had 2nd–3rd and 4th and above birth order (AOR = 1.25, 95 % CI = 1.06–1.46 and AOR = 1.26, 95 % CI = 1.05–1.51, respectively). According to the findings of our study, mothers who were working in sales and other sectors were less likely to commence breastfeeding within 1 h of birth as compared to mothers who were not working (AOR = 0.81, 95 % CI = 0.71–0.91). Also, mothers from the poorest households were less likely to commence breastfeeding early as compared to mothers from poorer (AOR = 1.41, 95 % CI = 1.15–1.73); middle (AOR = 1.61, 95 % CI = 1.27–2.04); richer (AOR = 2.01, 95 % CI = 1.57–2.58) and richest (AOR = 1.92, 95 % CI = 1.42–2.59) households. The following variables were not significantly related to EIBF; Mothers age at birth, mothers education, ANC visit, and birth type.

**Table 2:** Factors associated with early initiation of breastfeeding, Nigeria, 2013

Characteristic	Initiation of breastfeeding within 1 h of birth					
	Unadjusted OR			Adjusted OR		
	OR	95%CI	*p* value	AOR	95%CI	*p* value
Mother’s age at birth						
<=19	1.00			1.00		
20–24	1.18	(1.01–1.39)	0.042	0.95	(0.79–1.14)	0.547
25–29	1.31	(1.10–1.55)	0.002	1.10	(0.89–1.36)	0.359
30–34	1.33	(1.12–1.59)	0.001	1.08	(0.85–1.36)	0.535
> = 35	1.24	(1.04–1.47)	0.017	1.07	(0.84–1.38)	0.570
Mothers education						
No education	1.00			1.00		
Primary	1.36	(1.18–1.57)	<0.001	0.99	(0.85–1.16)	0.928
Secondary and above	1.57	(1.37–1.79)	<0.001	1.02	(0.86–1.21)	0.801
Birth order						
1st	1.00			1.00		
2nd–3rd	1.18	(1.02–1.35)	0.024	1.25	(1.06–1.46)	0.007
4th and above	1.10	(0.98–1.25)	0.121	1.26	(1.05–1.51)	0.013
ANC visit						
0	1.00			1.00		
1–3	1.53	(1.41–1.90)	<0.001	1.08	(0.89–1.30)	0.425
4+	1.64	(1.27–1.84)	<0.001	1.07	(0.91–1.27)	0.419
Place of delivery						
Home	1.00			1.00		
Health facility	1.65	(1.47–1.86)	<0.001	1.40	(1.22–1.60)	<0.001
Mode of delivery						
Caesarean section	1.00			1.00		
Vaginal delivery	1.88	(1.31–2.71)	0.001	3.08	(2.14–4.46)	<0.001
Size of child at birth						
Small	1.00			1.00		
Average	1.20	(1.04–1.38)	0.015	1.07	(0.93–1.24)	0.348
Large	1.45	(1.25–1.70)	<0.001	1.19	(1.03–1.39)	0.023
Birth type						
Twin/mutiple	1.00		1.00			
Singleton	1.37	(0.96–1.97)	0.078	1.33	(0.92–1.92)	0.133
Mothers occupation						
Not working	1.00		1.00			
Agricultural	1.04	(0.87–1.26)	0.650	0.90	(0.73–1.12)	0.359
Sales and others	0.87	(0.78–0.96)	0.008	0.81	(0.71–0.91)	0.001
Wealth Index						
Poorest	1.00		1.00			
Poorer	2.37	(1.90–2.95)	<0.001	1.41	(1.15–1.73)	0.001
Middle	2.51	(2.06–3.09)	<0.001	1.61	(1.27–2.04)	<0.001
Richer	2.00	(1.62–2.48)	<0.001	2.01	(1.57–2.58)	<0.001
Richest	1.49	(1.22–1.83)	<0.001	1.92	(1.42–2.59)	<0.001
Type of place of residence						
Rural	1.00			1.00		
Urban	1.62	(1.43–1.85)	<0.001	1.46	(1.23–1.75)	<0.001
Zone						
North West	1.00			1.00		
North East	1.81	(1.48–2.20)	<0.001	1.69	(1.38–2.07)	<0.001
North Central	2.53	(2.09–3.06)	<0.001	1.93	(1.56–2.39)	<0.001
South East	1.43	(1.13–1.82)	0.003	0.75	(0.56–1.01)	0.061
South South	2.23	(1.81–2.73)	<0.001	1.42	(1.12–1.81)	0.004
South West	1.09	(0.89–1.33)	0.401	0.56	(0.43–0.73)	<0.001

## Discussion

According to the WHO, 0–29 % prevalence of EIBF is considered as poor, 30–49 % as fair, 50–89 % as good and 90–100 % as very good [23]. Our result showed that the prevalence of EIBF EIBF in Nigeria is fair and stand at 34.7 %. The prevalence of EIBF observed in our study is much lower than what is observed in some African countries such as Ghana (46 %), Gambia (48 %) and Malawi (56 %) but much higher than in Pakistan (29 %), India (24.5 %) and China (23.2 %) [24]. These variations observed among countries might partly be attributed to cross-cultural difference in breastfeeding practice, for instance; Oche et al. [12] in their study found that the major reason for late initiation of breastfeeding in Kware, Northern Nigeria, was that most of the respondents believed colostrum was not pure and therefore could harm the infant [12]. Another study confirmed that a mothers decision to initiate and continue breastfeeding was determined by the perceived breastfeeding culture of her environment [25].

We found statistically significant associations between EIBF and the following variables; birth order, place of delivery, mode of delivery, size of child at birth, mothers occupation, household wealth index, place of residence and region. Similar findings were observed by Babatunde and Adebayo [26] in a trend analysis of EIBF rate in Nigeria between 1990 and 2008, however, their study did not control for mothers occupation, household wealth and size of child at birth.

In our study, mothers who delivered in a health facility were significantly more likely to inititate breastfeeding within 1 h of birth as compared to mothers who delivered at home. This is not surprising since many of the Primary Health Care Centers and hospitals in Nigeria have adopted the BFHI and the policy in those health care facilities is for the midwife or any other available skilled providers to encourage and assist in the process of achieving earlier initiation as defined by the BFHI target [27]. In a study done in Port-Harcourt, in the South South geopolitical zone of Nigeria, the authors observed that the presence of more than one delivery assistance as well as the presence of a breastfeeding trained delivery assistance in a health facility enhanced the mothers practice of EIBF [20]. However, the rate of EIBF among mothers who delivered in a health facility as reflected in our study is still low (42 %). Awi et al. in a study done at Port Harcout found an EIBF rate of 33.6 % among mothers who delivered in the hospital and had vaginal delivery and none among mothers who had caesarean section. They observed that routine labour ward practices and help recieved to initiate breastfeeding where the most important determinants of EIBF as compared to sociodemographic variables in a hospital setting [20]. In Nigeria, under staffing and over worked health care staff in health facilities might play a significant row in delaying EIBF. There is a need for a more detailed study on factors associated with lower rates of EIBF observed among mothers who delivered in a health facility.

The result of this study revealed that mothers who had vaginal delivery were more likely to inititiate breastfeeding within 1 h of birth as compared to mothers who had caesarean section and this finding is comparable to a study done by Rajan [28]. The difference between the two groups could be explained by the morbidity associated with ceasearean section, the effect of anesthesia, the emotional adjustment to the fact that the mother was unable to deliver normally and the exhaustion from a difficult labour that may have included many other intervention [20, 29].

We also observed a positive association between birth order and EIBF. In consonance, Lessen et al. [30] reported that previous breastfeeding experience was positively associated with both intention and initiation of breastfeeding, and the number of children was positively associated with initiation and inversely associated with intention to breastfeed.

In addition, this study showed that small sized babies were less likely to commence breastfeeding within 1 h of birth as compared to large sized infants and the finding was similar to what was observed in Turkey and Brazil [31, 32]. One of the possible explanation for this finding is that depending on birth weight, premature children have peculiarities and specific characteristics related to their own immaturity which may limit the abilities needed for breastfeeding within the first hour of life, such as good coordination of the suction-deglutition-respiration cycle and the breast-seeking reflex [33]. On the other hand, large sized babies may be perceive as healthy and fully matured with good coordination of the suction-deglutination-respiratory cycle and breast seeking reflex and therefore, may lead to initiating early breastfeeding [21].

Association between breastfeeding and socioeconomic status (SES) are complex as differing aspects of SES may be associated with knowledge, attitudes, experiences, and beliefs leading a woman to a particular infant feeding practice [34]. Among the measures of SES are; occupation, household wealth index and education [34, 35]. In our study, mothers occupation had a strong influence on EIBF. Mothers who were working in sales and other sector were less likely to initiate breastfeeding early as compared to mothers who were not working, this is in consonance with a previous study done by Fein and Roe [36] which revealed that full time employment decreased breastfeeding initiation and duration. On the other hand, the finding in this study was different from what was observed in Turkey [31]. The Turkish study showed that working status had no effect on initiation of breastfeeding, however, the association between maternal employment and initiation of breastfeeding has not been uniform and raises the need for further investigation.

Our results indicates that mothers from wealthier households were more likely to commence breastfeeding early as compared to mothers from poorest household. Similar result have being observed in a previous study which revealed that richer household wealth was associated with increased likelihood of EIBF [37].

Our findings showed that occupation and household wealth index were the significant SES variables that were associated with EIBF, maternal education on the other hand was not. This contradicts a study done in Nepal which indicate that both maternal education and occupation had significant effect on EIBF, whereas, household wealth index was observed not to be significantly related to EIBF [21]. A more detailed research might attempt to measure all aspects of SES to discern which dimenstions play’s the most important roles with regards to EIBF in Nigeria.

We observed that urban mothers were more likely to initiate breastfeeding within 1 h of birth as compared to mothers in rural area, this is in consonance with a previous study [38]. In rural areas, lower levels of education, incomes, lack of health insurance and access restrictions to health care may partly explain why breastfeeding initiation rates may differ for mothers in rural as compared to urban areas.

The odds of mothers inititiating breastfeeding within 1 h of birth were relatively low in the South South as compared to the North West, however the following geopolitical zones; North East, North Central, and South South had higher likelihood of EIBF as compared to the North West geopolitical zone. Such regional differences in breastfeeding practice have been observed in previous studies done in other countries [21, 37]. In Nepal for instance, the authors attributed the variations within regions to differing regional availability of infant formula and television advertisements, difficult terrain, poverty status and lower socioeconomic development indicators [21]. In Nigeria, the large geographic variation in EIBF (especially low rates in the North West and high rates in North Central region) need to be studied further.

### Policy and practice implication

Neonatal, infant and child mortality as well as malnutrition continue to be major health issues affecting Nigeria. Nigeria’s neonatal mortality rate stands at 37 deaths per 1000 live births [17] and EIBF rates in Nigeria as envidence in our study is below the WHO recommendation. A substantial increase in EIBF rate can be achieved in Nigeria by promoting hospital deliveries. Also, EIBF promotion programmes should target all mothers, but with special focus given to primiparous mothers, poor mothers, rural mothers and working mothers. In addition, breastfeeding interventions as they pertain to EIBF should cut across zones with more empasis to zones with lower rates of EIBF. Future research should focus on the low rates of EIBF observed among mothers who delivered in health facilities even though the rate of EIBF was observed to be considerable higher among mothers who delivered in health facilities as compared to mothers who delivered at home. Futhermore, zonal variations in EIBF need to be researched further.

### Strength and limitations

EIBF was based on self-report. This is a potential source of measurement bias in the outcome, where mothers may incorrectly recall when the child initiated breastfeeding. The findings are also based on cross-sectional data and therefore caution must be exercised in making causal influence of the identified determinants of EIBF. However, the study has a strength of being a nationally representive study.

## Conclusion

Overall, this research have shown that in Nigeria, breastfeeding initiation within 1 h of birth is not optimal and EIBF programmes and policies should focus on; rural mothers, working mothers, primiparous mothers, mothers with ceasarean deliveries, mothers with home deliveries and poor mothers.” It is also important that this interventions cut across geopolitical zones with more emphasis to zones with lower rates of EIBF.
